# A conceptual model of second language pronunciation in communicative contexts: Implications for children’s bilingual education

**DOI:** 10.3389/fpsyg.2023.1125157

**Published:** 2023-04-17

**Authors:** Youran Lin, Fangfang Li, Andrea A. N. MacLeod, Karen E. Pollock

**Affiliations:** ^1^Department of Communication Sciences and Disorders, University of Alberta, Edmonton, AB, Canada; ^2^Department of Psychology, University of Lethbridge, Lethbridge, AB, Canada

**Keywords:** second language, pronunciation, foreign accents, communication, children, bilingual education

## Abstract

Second language (L2) pronunciation patterns that differ from those of first language (L1) speakers can affect communication effectiveness. Research on children’s L2 pronunciation in bilingual education that involves non-English languages is much needed for the field of language acquisition. Due to limited research in these specific populations and languages, researchers often need to refer to literature on L2 pronunciation in general. However, the multidisciplinary literature can be difficult to access. This paper draws on research from different disciplines to provide a brief but holistic overview of L2 pronunciation. A conceptual model of L2 pronunciation is developed to organize multidisciplinary literature, including interlocutors’ interactions at three layers: the sociopsychological, acquisitional, and productive-perceptual layers. Narrative literature review method is used to identify themes and gaps in the field. It is suggested that challenges related to L2 pronunciation exist in communication. However, the interlocutors share communication responsibilities and can improve their communicative and cultural competencies. Research gaps are identified and indicate that more studies on child populations and non-English L2s are warranted to advance the field. Furthermore, we advocate for evidence-based education and training programs to improve linguistic and cultural competencies for both L1 speakers and L2 speakers to facilitate intercultural communication.

## 1. Introduction

Second language (L2) learners may acquire speech differently than first language (L1) speakers and produce speech with an accent (Munro, 1998). The concept of “foreign accents” is exonormative, as interlocutors look outward for perceived standard or prestige forms (Monfared, 2019). Thus, research on L2 pronunciation has implications for both communication efficacy and perception of identities.

Researchers from diverse disciplines have long been interested in L2 pronunciation (e.g., Lado, 1957; Giles, 1970; Munro and Derwing, 1995; Lippi-Green, 2011; Flege and Bohn, 2021). However, the research issues and approaches are often discipline-specific, which prevents a comprehensive understanding of the field and prevents researchers from studying a topic of interest in another discipline. Therefore, a new, transdisciplinary perspective that involves psychology, education, and linguistics will advance the field of speech acquisition.

Moreover, child L2 learners have been given less attention in research and practice. In the few discussions about child L2 speech acquisition, the target language was usually English (Derwing, 2020; Levis, 2020). Applying knowledge of L2 pronunciation learning to child learners of non-English is important for bilingual education programs across the world, especially for the ones where at least one of the target language(s) is not English, for example, the international language and indigenous language programs in Canada (Dicks and Genesee, 2017) and the Russian-Hebrew bilingual program in Isreal (Schwartz et al., 2016).

The goal of this paper is to provide a brief but holistic overview of the multidisciplinary literature on L2 pronunciation through a conceptual model and present implications for child bilingual learners of non-English languages. This encompassing model disentangles the interactions between L2 learners and their interlocutors in terms of their sociopsychological characters, linguistic experiences, and speech production and perception. This paper addresses researchers who are interested in pronunciation development in child bilingual speech acquisition. However, the model can be used by researchers of L2 pronunciation in general as a tool to organize their literature and situate their studies, and its implications provide new ideas to not only researchers, but also educators, practitioners, and policymakers.

Given the long-standing history and extensive breadth of the field, a scoping review would be unrealistic. Rather than reducing the scope, a narrative literature review methodology was adopted. The model was developed through extensive reading and discussion. Multidisciplinary literature was mapped onto this model to identify themes and gaps in research. This will point out the main issues of research and raise awareness of future research venues, especially the ones that tend to be neglected at multidisciplinary intersections. This paper will first introduce the three-layer conceptual model, then briefly review L2 speech research within each layer, and finally, present implications for child L2 learners of non-English languages through themes and gaps across the layers.

## 2. A conceptual model of L2 pronunciation in communicative contexts

**Figure 1:**
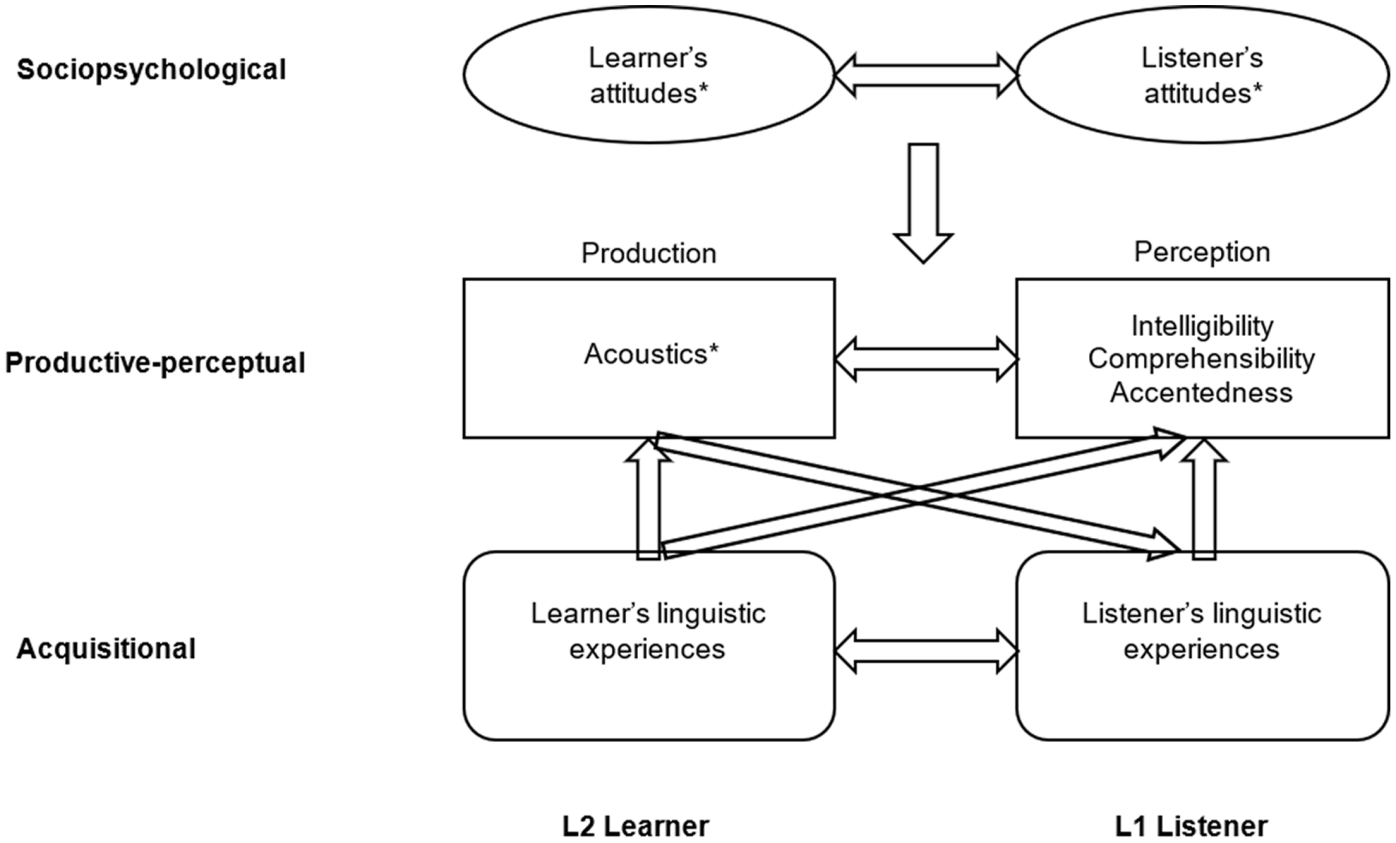
A three-layer conceptual model of L2 speech in communicative contexts. * The factor listed is only an example of the multidimensional information in the category.

Communication involves two or more people who convey and receive information. In the context of L2 oral communication, we will refer to them as the “L2 Learner” and “L1 Listener,” as if these were interlocutor roles or names. Such role assignment is oversimplified, as the interlocution is bidirectional, and communication also occurs among L2 speakers (Levis, 2020). However, such simplification allows us to discuss the speakers’ speech systems and social cultures and, with cautious comparisons, has the potential to generalize to diverse interlocutor groups. Therefore, we propose a model to understand the interactions between L2 Learners and L1 Listeners at and across three layers: the sociopsychological, acquisitional, and productive-perceptual layers (Figure 1).

The sociopsychological layer focuses on communicators’ attitudes toward L2 pronunciation, along with other individual and situational factors. The methods to understand attitudes include observation, interview, survey, and sociopsychological experiments (Giles, 1970; Lippi-Green, 2011). Understanding attitudes toward L2 pronunciation can help communicators become aware of biases and take mutual responsibility for communication (Clark and Wilkes-Gibbs, 1986).

The acquisitional layer addresses the roles of phonological (speech system) experience in pronunciation learning. For L2 Learners, there are a variety of theoretical models that discuss how L1 phonology impacts L2 learning (e.g., Best and Tyler, 2007; Flege and Bohn, 2021). In parallel, for L1 Listeners, the ability to listen to L2 speech is also impacted by their phonological experiences (Hau et al., 2020). Theories and studies in this layer provide frameworks for studies in speech production and perception (Flege et al., 2003) and have pedagogical implications.

The productive-perceptual layer is where L2 Learners and L1 Listeners are directly engaged in a “speech circuit (De Saussure, 1959)” and the characteristics of L2 Learners’ pronunciation act on L1 Listeners’ perception. Perception and production can be measured subjectively and objectively (Munro and Derwing, 1995; Flege et al., 2003), and their relationships can be identified through statistical analyzes and psychoacoustic experiments (Liu et al., 2014; Porretta et al., 2015). Such research can characterize L2 oral communication and suggest effective targets for pronunciation instruction (Trofimovich and Isaacs, 2012).

In this conceptual model, L2 speech production and perception are impacted by both sociopsychological and acquisitional factors. On the other hand, oral communication in the speech circuit can, in turn, affect interlocutors’ sociopsychological characters and linguistic experiences. Therefore, the layers are interrelated and the model does not proceed in a particular order.

## 3. The sociopsychological layer: Attitudes toward L2 pronunciation

Researchers of child L2 pronunciation should be mindful of the ecological complexity of communication. Many sociopsychological factors at the individual and situational levels contribute to L2 oral communication, including but not limited to personality (Rivers and Ross, 2020), willingness to communicate (Baran-Łucarz, 2014), emotional state (Suzukida, 2021), and cognitive workload (Farris et al., 2008). Moreover, communication is contextualized in a bigger picture of power dynamics and cultural stereotypes. This section will focus on L1 Listeners’ and L2 Learners’ attitudes toward L2 pronunciation at the individual and group levels.

### 3.1. L1 Listeners’ attitudes toward L2 pronunciation

L2 pronunciation that is accented does not necessarily cause ineffective communication, but it evokes the previously internalized attitudes toward certain groups (Derwing and Munro, 2015). L1 Listeners’ negative attitudes toward L2 pronunciation are widely reported (Lippi-Green, 2011). This can be due to (mis)beliefs about their own linguistic status and/or linguistic stereotyping of the L2 pronunciation.

English exemplifies the role of language status. As English is established as an international language, English users are often dichotomized into native and non-native speakers, which gives a higher status to the former. According to a survey (Roper Public Affairs, 2006), 75% of youths in the United States believed that English was the world’s most-spoken native tongue, and 38% considered speaking a foreign language “not too important.” In relation, the Inner-Circle varieties of English (e.g., American and British English) often enjoy privilege (Jenkins, 2013), although varieties of English are widely used in the Outer Circle (e.g., Singapore, India, Nigeria) as an official language and constitute the countries’ multilingualism (Kachru, 1990). The beliefs of language privilege impact the power dynamics between L1 Listeners and L2 Learners.

In addition, L1 Listeners’ attitudes toward accents can be related to social stereotypes against certain groups. According to Lippi-Green (2011), for example, attitudes toward French accents are positive for the majority of Americans, while many have negative reactions to Asian accents. Simply due to the stereotypes of how a member of a perceived group should sound, L1 Listeners’ speech perception may change, which is referred to as reversed linguistic stereotyping (Kang and Rubin, 2009). For example, Babel and Russell (2015) found that L1 Listeners had more difficulty transcribing the English speech produced by Chinese Canadian speakers when photos of their faces were presented. On the other hand, even before acquiring linguistic stereotypes, preschool-aged children already show selective trust in native-accented informants, which indicates that children are more invested in learning from members of their own cultural groups (Kinzler et al., 2011). This provides insights into how the preference for native accents is formed.

However, negative attitudes do not necessarily lead to communication failures, since their influence is mediated by communication strategies. Lindemann (2002) paired Korean English learners with two groups of English-L1 Listeners who had negative and positive attitudes to Koreans, respectively, for an interactive task. The tasks were completed successfully as long as L1 Listeners actively provided feedback. In contrast, the tasks failed when the L1 Listeners were avoidant, refusing to provide any crucial feedback and completely attributing the communication difficulties to L2 Learners. This suggests that communication can be improved through interventions on communication strategies even when attitudes are not directly addressed.

### 3.2. L2 Learners’ attitudes toward L2 pronunciation

L2 Learners have various attitudes to foreign accents. Some learners, especially those who are in the Expanding Circle (Kachru, 1990) and learn English as a foreign language, admire native speech as the perfect example and ascribe higher status to the Inner-Circle varieties (Carrie, 2017). For example, Japanese and Korean English learners disapproved of their varieties of English and prioritized “nativeness” in their English pronunciation (Tokumoto and Shibata, 2011).

In contrast, L2 Learners accept their accents better when they perceive themselves as users of a legitimate variation of the language (Lippi-Green, 2011), for example, English speakers from the Outer Circle. In comparison to the Japanese and Korean learners aforementioned, Malaysian English learners valued message conveyance more than nativeness (Tokumoto and Shibata, 2011). In addition, L2 Learners may have positive attitudes toward their accent when it marks their identity as desired. For example, among French-English bilinguals in Québec, stronger non-native accents in English were associated with sociopolitical affiliation to the group (Gatbonton and Trofimovich, 2008).

Sociopsychological factors play a role in L2 Learners’ pronunciation learning outcomes (Sardegna et al., 2014). Saito et al. (2017) found that L2 Learners who were able to improve their pronunciation over one academic semester tended to show motivations to learn English as a long-term resource. These students produced L2 speech that was easier for listeners to understand, even though their pronunciation might not be native-like. Nonetheless, the contribution of attitudes and motivations of learning L2 pronunciation should be examined with caution. Sardegna et al. (2018) suggested that although L2 Learners’ strong motivation was associated with more efforts to improve L2 pronunciation, it also predicted negative emotions with regard to L2 pronunciation, which might in return barrier their oral communication.

### 3.3. Summary of the sociopsychological layer

To summarize, a preference for native pronunciation occurs at young ages, and negative attitudes toward non-native pronunciation can impede L2 communication. Researchers advocate for more inclusive attitudes toward L2 pronunciation.

Despite the rich discussion on attitudes toward L2 pronunciation, we identify the languages involved in research as a gap in this layer. Literature is rich in the attitudes toward accented English, but less is known about the attitudes toward L2 pronunciation of non-English languages (e.g., Marx, 2002, an English-L1 learner of German, reflected on their German accent and identity). In some non-English languages, research focuses on accents of native varieties. For example, Chong and Tan (2013) investigated Singaporean Chinese youths’ attitudes toward the Beijing, Taiwan, and Singapore varieties of Mandarin; Lindberg and Trofimovich (2020) examined French-L2 Learners’ attitudes toward the European and Québec varieties of French. That being said, our research was mainly on the body of Literature that was written in English, which limited our access to literature in other languages. It remains unclear whether our knowledge of attitudes toward accents in English are equally applicable to other languages, as English has the special status of an international language.

Researchers of child L2 pronunciation should understand that even for young learners, their L2 learning and communication are impacted by sociopsychological factors. This becomes especially complex and important when the children’s L1 is a high-status language, for example, English-speaking children learning an international language through bilingual education. In addition to the sociopsychological layer, L2 pronunciation learning and communication are also impacted by the specific L1-L2 pair and the interlocutors’ experiences in these languages.

## 4. The acquisitional layer: The impacts of linguistic experiences

Researchers of child L2 pronunciation should understand the mechanisms of learning new phonological systems so they know what learning outcomes to expect given a specific L1-L2 pair. There are several impactful L2 speech acquisition theories, each with its own assumptions and predictions, which can be challenging to access for researchers who are first attempting to tackle L2 pronunciation issues. This section will introduce important theories comparatively to help researchers understand how L2 pronunciation learning is impacted by L2 Learners’ phonological experiences. We then argue that L1 Listeners are parallelly biased by their linguistic experiences when communicating with L2 Learners.

In the past 70 years, L2 speech acquisition models have evolved from the Contrastive Analysis Hypothesis (CAH, Lado, 1957) and the Critical Period Hypothesis (CPH, Lenneberg, 1967) to the Perceptual Assimilation Model of L2 Speech Learning (PAM-L2, Best and Tyler, 2007), the Speech Learning Model (SLM-r [revised], Flege and Bohn, 2021), and the Second Language Perception Model (L2LP-r, Van Leussen and Escudero, 2015). In this process, at least three themes have been discussed: (a) the mechanisms of L2 speech acquisition, (b) the roles of non-phonetic information, and (c) the bidirectional interactions between L1 and L2. See Table 1 for a summary of these models and two infant speech development models in comparison, i.e., Native Language Magnet theory [NLM-e (expanded), Kuhl et al., 2008] and Processing Rich Information from Multidimensional Interactive Representations (PRIMIR, Werker and Curtin, 2005).

**Table 1:** A summary of the speech development theories reviewed.

	CPH	CAH	PAM	SLM	L2P2	NLM	PRIMIR
Perception/production	Both	Both	Perception	Both	Perception	Perception	Perception
Learning mechanism	Implicit, language-specific	Behavioristic	Ecological	Psychoacoustic, statistical	Connectionist, statistical	Statistical	Statistical
Identical mechanisms for L1 and L2?	No	Yes	Yes	Yes	Yes	No	Not discussed
Why is L2 speech learning challenging?	Neural maturation	Negative transfer of L1	Perceptual assimilation	Perceptual assimilation; limited input	Weak L2 connections	L1 Neural commitment	Not accessing the acoustic cues
Object of perception	Not discussed	Not discussed	Articulatory gestures	Phonetic distance	Words (meaning errors)	Acoustic information	Multidimensional information
Level of analysis	Not discussed	Phonemic	Phonemic	Allophonic	Multiple levels	Prototypes	Multiple levels
Non-phonetic factors	Not the focus	Not the focus	Not the focus	Age of learning, L2 input	Word recognition	Social interaction	Linguistic and social information
L2-to-L1 effects?	No	No	Not the focus	Yes	Yes, when the layers are situated interactively	Not the focus	Not the focus

### 4.1. The mechanisms of L2 speech acquisition

L1 speech is usually acquired rapidly and effortlessly in infanthood (Kuhl et al., 2008). However, L2 speech learning can be protracted and effortful for L2 Learners who started at an older age, and the speech learning outcome can be inaccurate and accented (Flege, 1995). Theorists discussed the cause of such differences. In the mid-20th century, CPH proposed that young children learn speech through mechanisms that are specialized for language learning, but older learners lose such abilities due to neural maturation (Lenneberg, 1967). On the other hand, CAH regarded language learning as habit formation. When the speech systems are different, L1 habits negatively transfer to L2 learning (Lado, 1957; Wardhaugh, 1970). CAH is powerful in predicting L2 speech learning difficulties by comparing the speech systems of L1 and L2.

However, different speech learning occurred as early as among young school-aged bilingual children (Netelenbos et al., 2016; Yang and Fox, 2017), which challenged the notion of “earlier is better.” Since the 1990s, perception-based theories have developed, represented by PAM, SLM, and L2LP. According to these theories, speech learning mechanisms remain unchanged across the lifespan. Nonetheless, L2 Learners’ perception is attuned by their L1. This hinders the acquisition of L2 pronunciation but does not completely block it (MacLeod and Stoel-Gammon, 2010). However, these theories have different views on the specific mechanisms of L2 speech perception.

PAM predicts how naïve listeners or new learners perceive a *contrastive pair* (a pair of sound categories that differentiate word meanings) in L2 based on how they are assimilated to L1 categories. For example, if two L2 sounds are perceived as exemplars of two different L1 categories, PAM predicts good discrimination of this pair; but if both sounds are perceived as equally good exemplars of the same L1 category, PAM predicts poor discrimination. To establish a new category in L2, learners need to detect the gestural features of the L2 sounds and contrast them in *minimal pairs* where the sounds differentiate word meanings in the L2 (Best and Tyler, 2007).

One of the PAM’s advantages is its specific predictions of challenging targets, which guides research and pedagogical practice. Moreover, it is explanatorily powerful as it has been generalized to suprasegmental elements (So and Best, 2010). However, when applying PAM, researchers should understand that (a) PAM analyzes gestural features that are contrastive, but not the phonetic or allophonic details of speech production (e.g., /t/ in “cat” can be released or unreleased, but such phonetic differences do not change the meaning); and (b) PAM’s intent is to account for the perception of new learners instead of experienced learners (Best and Tyler, 2007).

Different from PAM, SLM is interested in the establishment of new phonetic categories in L2, which is based on the phonetic dissimilarity between the L2 sound and its closest L1 counterpart (Flege, 1995). Therefore, SLM’s learning objects are sounds instead of sound pairs, and the analysis is phonetic. Flege (1995) stated that L2 speech acquisition was predicted by phonetic dissimilarity and age of onset. In SLM-r (Flege and Bohn, 2021), age of onset was respecified as a macro-variable related to the quantity and quality of L2 speech input. Moreover, SLM initially focused on experienced learners, while SLM-r embraced an unchanged mechanism of speech acquisition: statistical learning (see Kuhl et al., 2008 for statistical learning in infant speech acquisition). Therefore, it aims to account for the full process of L2 speech acquisition.

Despite the evolution of SLM-r, researchers should understand: (a) according to SLM, L2 speech acquisition is impacted by perceived phonetic dissimilarity, while a measurement of such dissimilarity remains undefined (Flege and Bohn, 2021); (b) the quantity and quality of L2 speech input have not been operationalized (although Flege, 2021, proposed a method, it was a self-reported survey that heavily focused on L2 Learners’ output instead of input); and (c) SLM discusses pronunciation deviances from the native norm, which is not fully compatible with the focus on intelligibility in L2 pronunciation education (Munro and Derwing, 1995; Levis, 2020).

In contrast to PAM and SLM, L2LP is interested in the *connections* between the acoustic, phonological, and lexical levels (Van Leussen and Escudero, 2015). For new L2 Learners, the acoustic-phonological connection is inherited from L1, so the weak connection in L2 constrains the learner from choosing the appropriate path. As L2 experiences increase, the appropriate L2 connections are strengthened. Meanwhile, the L1-inherited path is weakened whenever a meaning error (misunderstanding in communication) occurs, and as a result, a more plausible path is accessed.

L2LP uses computational models to simulate learning, which allows for quantifiable and testifiable predictions. However, when applying L2LP, some caveats should be considered: (a) several parameters in the computational model are arbitrarily set up, which might not fully represent reality; and (b) the results of simulated learning are not ground truths and need to be tested empirically (Van Leussen and Escudero, 2015).

In summary, perception-based theories argue that L2 speech learning is hindered but not blocked by L1-attuned perception. Through them, researchers can understand how L2 Learners’ linguistic experiences impact their L2 pronunciation acquisition and predict specific challenges in learning by examining their L1 and L2 phonological systems.

### 4.2. The roles of non-phonetic information

Non-phonetic information, such as lexical and social-interactive information, is important in speech acquisition (Clark and Wilkes-Gibbs, 1986; Werker and Curtin, 2005; Kuhl et al., 2008). The following paragraph will compare how perception-based theories consider non-phonetic information in L2 pronunciation acquisition.

PAM focuses on contrastive pairs and involves a lexical perspective by nature. Furthermore, PAM predicts that there is more communicative pressure to learn L2 sound pairs that involve high-frequency words, dense phonological neighborhoods, and/or importance in social communication (Best and Tyler, 2007). However, PAM does not make specific hypotheses about these factors. On the other hand, SLM-r focuses on the distribution of phonetic information in L2 input (Flege and Bohn, 2021). Such a distributional perspective potentially involves word frequency, learner factors, and social interactions. However, these factors are not yet unpacked in SLM-r. Different from the other two models, L2LP argues that learning is driven by lexical information (Van Leussen and Escudero, 2015). Whenever L2 pronunciation causes a misunderstanding, the L2 Learner will attempt to improve their speech perception until a more plausible path is accessed. This mechanism of using multidimensional information in speech learning is similar to PRIMIR’s proposals about infant speech acquisition (Werker and Curtin, 2005).

To summarize, L2 speech acquisition models take linguistic, non-phonetic (e.g., lexical) information into account to different degrees. However, none of them directly addresses the effects of the linguistic-external factors in the sociopsychological layer. Sociopsychological factors such as language status, language attitudes, and motivation play an important role in L2 speech learning and communication (Lindemann, 2002; Meziane and MacLeod, 2017; Saito et al., 2017; Sardegna et al., 2018) and should be further incorporated into the theories mentioned in the acquisitional layers.

### 4.3. The bidirectional interactions between L1 and L2 speech systems

For L2 Learners, the interaction between two languages is not unidirectional from L1 to L2. Instead, the L2 phonology can also influence their L1. PAM focuses on new L2 Learners and pays limited attention to L2 effects. In contrast, SLM and L2LP discuss L2-to-L1 influences.

SLM has a radical view on L2-to-L1 influence. It believes that L1 and L2 sounds occupy the same phonetic space, therefore L2 effects are immediate and inevitable. When an L2 category is not established, the neighboring L1 categories are assimilated because they are perceptually linked. When an L2 category is established, the L1 categories are dissimilated to maintain phonetic contrast (Flege and Bohn, 2021). Some evidence supports this hypothesis (Flege et al., 2003), but other work shows that L2 effects are more complicated, impacted by language dominance and communicative partners (de Leeuw et al., 2010; Yang and Fox, 2017).

L2LP accounts for such complexity, at least in part, by assuming different models in simulated learners. In a bottom-up model, i.e., when the acoustic, phonological, and lexical strata are separated, L1 phonetic categories are retained. On the contrary, when these aspects are interactive in one stratum, learners will eventually adopt the L2 system and lose the L1 system (Van Leussen and Escudero, 2015). The authors suggest that the bottom-up model resembles adult learning that rarely reaches native-like speech. This implies that the interactive model is in line with younger learners who experience L1 attrition (e.g., Yang and Fox, 2017) and provides an insight that L2-to-L1 effects may be stronger when the L1 phonological representations are not entrenched in young children.

It is clear that L2-to-L1 effects exist and are multifaceted. Empirical evidence shows that L2 can cause both segmental and suprasegmental changes in L1 (e.g., Flege et al., 2003; Bergmann et al., 2015). Research should pay continuous attention to L2-to-L1 influence. This is a particularly relevant real-life issue for bilingual children in immigration contexts as it has implications for L1 attrition.

### 4.4. L1 Listeners’ speech perception

Previous sections introduced how L2 Learners’ perception is attuned by their linguistic experiences. Given that the speech learning mechanism, i.e., statistical learning, remains unchanged across lifespan (Flege and Bohn, 2021), we compare L1 Listeners’ perception parallelly to L2 Learners’. This means L1 Listeners’ perception is also attuned by their L1 phonology and experiences perceptual “learning” when encountering a new speech system, i.e., perceptual adaptation (Hau et al., 2020). L1 Listeners adapt to accented speech rapidly within 1 minute (Clarke and Garrett, 2004) and draw upon non-phonetic information to facilitate understanding (Cooper and Bradlow, 2016). Perceptual adaptation occurs in not only adults but also in school-aged children (Hu, 2021) and generalizes to novel talkers and novel accents (Baese-Berk et al., 2013). Such perceptual learning sets the foundation to train L1 Listeners to understand accented speech. Derwing et al. (2002) found that instructions about the accents of a certain language group not only facilitated a better comprehension but also improved L1 Listeners’ attitudes.

### 4.5. Summary of the acquisitional layer

For L2 Learners, several themes were discussed by L2 pronunciation acquisition models, including the learning mechanisms, the roles of non-phonetic information, and bidirectional interactions between L1 and L2. A few research gaps are identified: First, most theories focused on speech sounds but not suprasegmental features (except for PAM, So and Best, 2010). Second, more evidence in children is needed to account for the full process of L2 speech acquisition indicated by SLM-r (e.g., Netelenbos et al., 2016; Menke, 2017; Meziane and MacLeod, 2017; Nance, 2020). Third, theories should further account for the effects of language-external factors such as social interactions, motivations, and attitudes. For L1 Listeners, research shows that relevant linguistic experiences (i.e., exposure to accented speech) facilitate perceptual adaptation and improve cultural competence. More research is expected to facilitate effective communication on the end of L1 Listeners who have the need to better understand accented speech.

## 5. The productive-perceptual layer: Perceptual measurements of L2 speech and their acoustic sources

Researchers of child L2 pronunciation should be familiar with the common measurements of L2 pronunciation. This section introduces two types of measurements based on the acoustics of speech production and L1 Listeners’ perception, respectively. These two measurements are important because interlocutors’ interaction ultimately happens in the “speech circuit (De Saussure, 1959)” when the speech is produced and perceived. It is noteworthy that such interaction is a multimodal phenomenon, where gestures, facial expressions, and environments all play a role. Among them, auditory signals have attracted the most attention, and acoustic measurement is chosen as one method to describe speech production.

### 5.1. Perceptual measurements of L2 speech

L2 pronunciation used to be perceptually measured by “accuracy” as if it was unidimensional (e.g., Olson and Jay Samuels, 1973; Suter, 1976). Munro and Derwing (1995) divided L1 Listeners’ perception of L2 pronunciation into related but distinctive aspects, including intelligibility, comprehensibility, and accentedness. Comprehensibility is defined as the ease of understanding L2 speech, while intelligibility is the extent to which listeners can understand the message. Therefore, comprehensibility is usually rated on a scale, and intelligibility can be calculated through the percentage of words recognized (Fayer and Krasinski, 1987; Munro and Derwing, 1995). In contrast, accentedness is defined as the perceived difference compared with a reference accent and is usually rated on a scale (Southwood and Flege, 1999). By teasing them apart, Munro and Derwing (1995) argued that the goal of L2 pronunciation learning was not reduced accentedness, but increased intelligibility and comprehensibility.

Researchers often consider L1 Listeners as a homogeneous population and measure L2 pronunciation through their perception (Munro and Derwing, 2020). The literature review in the sociopsychological and acquisitional layers suggests that L1 Listeners’ perception is biased by their attitudes and linguistic experiences (Kennedy and Trofimovich, 2008; Shintani et al., 2019). Therefore, it is important to be aware of these confounding factors when using perceptual measurements (Lindemann and Subtirelu, 2013). Researchers should choose carefully what speaker information to disclose: One possible option is to conceal identifying information to avoid biases based on linguistic stereotyping. The other is, contrariwise, to incorporate as much information as possible to resemble authentic communicative situations. Moreover, perceptual judgments should be paired with language background questionnaires and attitudinal measurements to account for biases (Dewaele and McCloskey, 2015; Munro and Derwing, 2020). In addition, it is important to use acoustic measurements to validate L1 Listeners’ perception and provide phonetic details (Lindemann and Subtirelu, 2013).

### 5.2. Acoustic cues of L1 Listeners’ perception of L2 speech

The source of L1 Listeners’ perception of L2 pronunciation is partly contained in the acoustic signals of L2 speech production. It is intuitive to use acoustic measurements to describe L2 pronunciation. However, researchers should be cautious of using acoustic data alone as not all dimensions of acoustic deviances are equally predictive of perceptual differences (Munro and Derwing, 2020). Nonetheless, acoustic measurements can be used in combination with L1 Listeners’ perception to validate the latter. In addition, such a combination can identify the acoustic dimensions that are important for intelligibility and, in turn, specify targets for efficient L2 instruction (Schertz and Clare, 2020).

As early as Ryan (1973) called for a production-based measurement of L2 pronunciation. Flege (1984) cross-spliced speech samples of English speakers and French speakers and found that L1 Listeners could detect non-native speech accurately. The study did not measure the acoustics directly, but this was an early experimental attempt to address the relationships between acoustic deviations and listener perceptions. In a later study, Flege et al. (2003) used L1 Listener judgment and acoustic measurements to measure English [e^ɪ^] and Italian [e] produced by Italian-English bilingual speakers. Regressions revealed that the difference between the first and second formants accounted for most of the variation in listener perception. Flege et al. (2003) was different from Flege (1984) in that it took multiple acoustic measurements and explored acoustic variables’ effects on perception.

Similar studies were expanded to more speech features in a variety of languages. Some showed that the perception was mainly impacted by spectral features (e.g., Wayland, 1997), while others suggested that temporal features played a role (e.g., Porretta et al., 2015; see Derwing and Munro, 2015 for a summary). However, in such studies, researchers could not establish causal relationships between acoustic deviances and perception or guarantee that the acoustic variables included were exhaustive (Porretta et al., 2015). This pointed to two directions of studies: (a) manipulated acoustic properties to establish causality and (b) a more extensive list of acoustic measurements.

Acoustic properties can be synthetically manipulated to verify causal relationships between acoustics and perception. For example, Liu et al. (2014) observed that L2 Learners might use duration as a cue to differentiate lax and tense vowels in production. To prove this hypothesis, they equalized the duration of L2 Learners’ productions to find that intelligibility was reduced. In contrast with how Liu et al. (2014) removed one dimension of acoustic variance, acoustic cues can be varied to form a continuum. Chan et al. (2017) manipulated spectral features gradually and found that the frequencies of vowel formants were a primary cue for the perception of L2 speech.

On the other hand, recent studies included larger sets of acoustic measurements. Idemaru et al. (2019) examined the impacts of vowel, consonant, rhythm, pitch, and fluency properties in Japanese-L2 Learners’ productions. Pitch errors were most predictive of accentedness for both English-and Mandarin-L1 learners of Japanese. L1-specific patterns were further identified. For example, vowel properties were predictive of English-L1 Learners’ accentedness perceived by Japanese L1 Listeners, while consonant properties were predictive of Mandarin-L1 Learners’ perceived accentedness. The large inventory of acoustic measurements provides a foundation to compare learners from a variety of language backgrounds and to explore the crucial acoustic factors for a specific pair of L1 and L2.

### 5.3. Summary of the productive-perceptual layer

In summary, L2 Learners’ production and L1 Listeners’ perception are the two ends of the speech circuit. Researchers use them to measure L2 pronunciation and examine the relationships between these two types of measurements. Such research attempts to validate the perceptual measurements, rank the gravity of acoustic deviances, and ultimately facilitate effective L2 pronunciation learning. Therefore, productive-perceptual studies have implications for speech acquisition in L2 pedagogy.

A few future directions that already emerged can be further explored in this layer. First, research generalizability in different languages should be considered. On one hand, more productive-perceptual studies in non-English languages are needed. Such studies may provide insights into the universality and uniqueness of acoustic correlates of L1 Listeners’ perception in different languages and guide pronunciation instruction in these languages (Porretta et al., 2015). On the other hand, a more thorough list of speech features can be developed with the potential to be used in any given L1-L2 pair (Idemaru et al., 2019).

Second, discourse studies are warranted to mimic more realistic communicative situations. The early productive-perceptual studies elicited single words (e.g., Flege et al., 2003), which could not provide a valid evaluation of L2 Learners’ speech. In recent studies, learners were prompted to produce sentences (e.g., Idemaru et al., 2019). However, these studies are still limited to laboratory environments. Future studies can look into acoustic and perceptual measurements of conversational speech and examine the impacts of linguistic and sociopsychological information to increase ecological validity and better represent real-life communication.

## 6. Synthesis across layers

We have proposed a three-layer conceptual model of research on L2 pronunciation in communicative contexts between L2 Learners and L1 Listeners, which includes sociopsychological, acquisitional, and productive-perceptual layers. Through a narrative literature review, we mapped existing research onto the model and identified research themes and future directions within each layer. Here we will discuss the interconnections across layers and some forward-looking ideas for children’s pronunciation acquisition of a non-English L2.

### 6.1. Interconnections between the layers

The layers of the model are interconnected, therefore the model does not proceed in a certain order. In the sociopsychological layer, both L1 Listeners and L2 Learners may have negative attitudes toward L2 speech. The attitudes can interact with the productive-perceptual layer. An example is reversed linguistic stereotyping (Kang and Rubin, 2009), where L1 Listeners experience perceptual difficulties solely due to the perceived group membership of the speaker.

In the acquisitional layer, L2 Learners’ perception is L1-attuned (MacLeod and Stoel-Gammon, 2010). Parallelly, L1 Listeners’ perception is also attuned by their L1, and they experience perceptual “learning” (adaptation) when exposed to L2 speech (Hau et al., 2020). Linguistic experiences of L2 speech can improve L1 Listeners’ knowledge of L2 pronunciation and improve intelligibility in the productive-perceptual layer (Kennedy and Trofimovich, 2008). Furthermore, knowledge and experiences of L2 speech improve L1 Listeners’ attitudes toward accented speech in the sociopsychological layer (Derwing et al., 2002).

In the productive-perceptual layer, perceptual and acoustic measurements of L2 pronunciation are also interconnected with the other two layers. As for perceptual measurements, L1 Listeners’ attitudes and perceptual adaptation may confound their perception. As for acoustic measurements, the acoustic features were usually chosen based on language-specific comparisons, guided by the theoretical models in the acquisitional layer.

### 6.2. A theme across layers and the need for intervention

The common theme across layers can be summarized as follows: L2 Learners are often faced with difficulties in L2 communication, but both L1 Listeners and L2 Learners can share a mutual responsibility to improve communication effectiveness (Clark and Wilkes-Gibbs, 1986). L2 pronunciation itself is not the cause of difficulties in communication, but the difficulties related to it should not be downplayed or ignored. L2 Learners are faced with real difficulties: Their perception has been attuned by their L1, which causes difficulties learning the new phonological system. In addition, L2 oral communication is affected by negative attitudes of both L1 Listeners and L2 Learners. To address these issues, interlocutors should share the mutual responsibility of communication and be supported to improve communicative skills.

For L1 Listeners, limited listening skills and prejudicial attitudes can cause hardship in communication. This can be addressed by improving perceptual adaptation and cultural competence (Derwing et al., 2002). Proposals to mitigate L1 Listeners’ attitudes and listening skills have been questioned, with a hesitation rooted in the belief that interventions aimed at L1 Listeners are too effortful and unfeasible, and that L2 oral communication is primarily a problem for L2 Learners.

However, perceptual adaptation to L2 speech can happen rapidly in both adults and children, and the learning outcomes can generalize to other accents (Clarke and Garrett, 2004; Baese-Berk et al., 2013; Hu, 2021). In addition, L1 Listeners’ negative attitudes can be confronted and improved through training sessions (Kang et al., 2015), and such improvements can result in enhanced perception of L2 speech (Cooper et al., 2020). Therefore, interventions that aim to address L1 Listeners’ attitudes and perception are feasible, and they are necessary at least for the groups that need to communicate with L2 Learners frequently, for example, educators, university students, healthcare providers, and public servants. Subtirelu and Lindemann (2016) proposed three aspects of L1-Listener interventions: (a) improving attitudes, (b) familiarizing with L2 pronunciations, and (c) developing communicative strategies. Future research can refer to these principles in their intervention designs.

Similarly, L2 Learners’ speech proficiency and cultural competence can be improved to facilitate effective communication. In terms of speech proficiency, L2 speech acquisition is a dynamic process, and the outcomes can be improved as the L2 speech input quantity and quality increase (Flege and Bohn, 2021). Moreover, researchers investigated the acoustic cues of perceived unintelligibility (e.g., Idemaru et al., 2019), which can be translated into pedagogical targets in L2 pronunciation teaching and learning. In terms of attitudes, L2 Learners’ attitudes are closely related to the language ideologies in their L2 classrooms. Unfortunately, L2 speech education often serves to ossify negative attitudes toward foreign accents (Lippi-Green, 2011). Negative feelings toward certain accents were reported among L2 teachers (Munro et al., 2006). Meanwhile, the teaching model still tends to be exonormative, i.e., British and American Englishes are often positioned as a standard (Monfared, 2019).

Fortunately, on the other hand, intervention programs have been designed in teacher education and English-L2 classes to mitigate language attitudes. For example, preservice English teachers’ attitudes improved after being exposed to diverse Englishes and practicing self-reflection (Ates et al., 2015). For L2 Learners, Korean university students participated in an extracurricular project to interview diverse English users (Lee, 2019). Students reported that the lack of exposure to diverse Englishes caused their preference for American English, while the authentic communicative situations brought attitudinal changes. Different from this project-based design, pedagogies in a university in China designed a structured program on language attitudes, including four steps: eliciting attitudes, deconstructing stereotypes, reconstructing open attitudes, and developing solutions to communication problems (Zheng and Gao, 2017). Almost half of the students embraced the concept of World Englishes after the intervention, while others remained ambivalent or conservative, indicating the necessity of continuous efforts and authentic communicative experiences to alternate the entrenched attitudes. Comparing these projects with Subtirelu and Lindemann’s (2016) proposal aforementioned for L1-Listener intervention, it seems that the L2-Learner intervention should also include at least three aspects: (a) reconstructing attitudes, (b) familiarizing with a variety of pronunciations in the target L2, and (c) developing communicative strategies.

**Figure 2:**
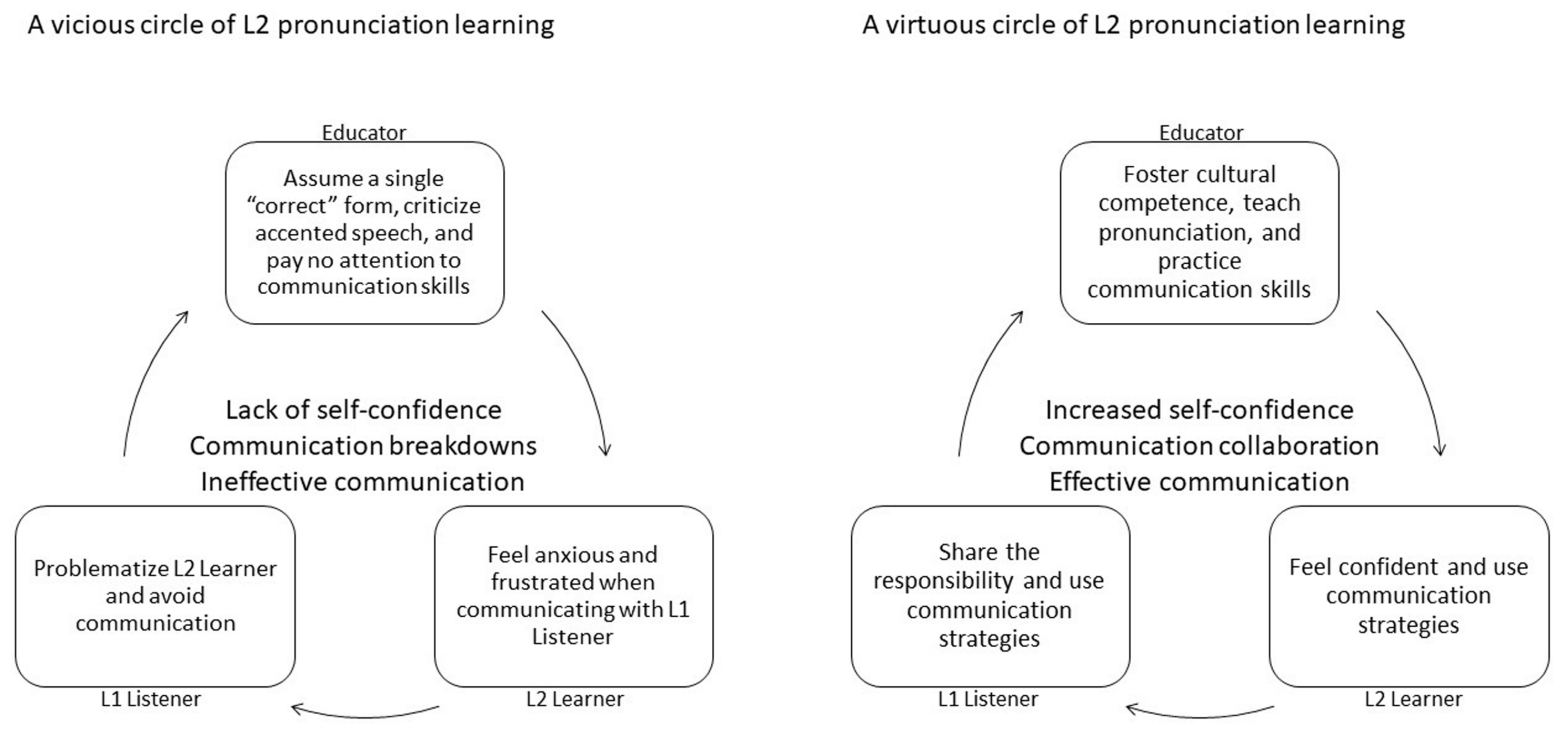
Attitudinal aspects of L2 speech in communication: vicious and virtuous circles.

Synthesizing the evidence, the interactions between Educators, L2 Learners, and L1 Listeners are illustrated in Figure 2. In a vicious circle, L2 Learners form negative attitudes toward foreign accents in the classroom, feel anxious during the communication with the L1 Listener, and are frustrated by L1 Listeners’ avoidant behaviors. On the other hand, when Educators foster open attitudes toward L2 pronunciation, L2 Learners feel prepared with improved pronunciation and communicative skills, and L1 Listeners are ready to adapt to L2 pronunciation, a virtuous circle can occur in L2 communication.

### 6.3. Research gaps and implications for child bilingual education

Research has addressed most of the layers in the model and their interconnections. However, several cross-layer gaps can be further considered to advance the field of L2 speech acquisition.

First, the vicious/virtuous circle that involves Educators, L2 Learners, and L1 Listeners (Figure 2) can and should be addressed with interventions. Recent efforts have been made to improve the cultural competence of L1 Listeners and L2 Learners, but effectiveness studies are warranted to understand what program designs are of merit. Moreover, most of the intervention programs are aimed at adolescents or adults, while stereotypes against L2 pronunciation can occur in preschool-aged children (Kinzler et al., 2011). Therefore, it remains unclear whether it is necessary and feasible to intervene in language attitudes at a younger age, especially for immigration children and their peers. Evidence is needed on whether and how bilingual education plays a role in dismissing linguistic stereotypes. Qualitative evidence shows that bilingual education in a minority language empowers students through cultural confirmation, nourishing positive self-identity, and encouraging transculturation (e.g., Wu, 2005). Little is known about how such cultural competency translates to positive attitudes toward diverse pronunciation.

Second, more research in non-English languages is needed. In this paper, we tried to include evidence from other languages (e.g., Chong and Tan, 2013; Idemaru et al., 2019; Lindberg and Trofimovich, 2020), but our access to literature in different languages was limited. However, with the dominant position of English, it is not surprising that most of the research on L2 pronunciation focused on English as the target L2 (Derwing and Munro, 2015). The issues of pronunciation in English are relevant to other languages (Levis, 2020), but learners’ motivation and speech input can be different when they are English speakers learning a non-English language. Therefore, studying L2 speech in non-English languages can help understand the generalizability of research, identify different perspectives on pronunciation in different cultures, and help the learners improve their oral communication.

Third, compared with the rich literature on adult L2 pronunciation acquisition, less attention is given to child learners. Derwing (2020) pointed out that this is in part because child L2 Learners’ pronunciation is usually thought to be native-like or, at least, intelligible. They discussed the L2 pronunciation difficulties in immigrant children and methods to facilitate their pronunciation learning of the societal majority language. However, little is known about how children learn the pronunciation of a minority language. For example, children who learn French as an L2 in Canada through immersion education showed non-native-like patterns in their consonants (Netelenbos et al., 2016), but in a Spanish-English bilingual school in the States and a Gaelic-medium school in Scotland, children’s pronunciation converged despite whether they were exposed to the minority language at home or not (Menke, 2017; Nance, 2020). It seems that the high-quality interaction with native-or heritage-speaking peers played a role in the pronunciation acquisition of a minority L2. To verify this observation and understand other learning factors, we advocate for more research that focuses on the L2 pronunciation acquisition of children who are learning a minority language of the society, in addition to the immigration children who are learning the majority languages.

## 7. Conclusion

Despite the limitation that a review paper cannot comprehensively cover the literature across multiple disciplines and a long history, this paper provides a narrative review on L2 pronunciation that focuses on the L1 Listener and L2 Learner’s interactions at the sociopsychological, acquisitional, and productive-perceptual layers. Through this review, we propose several “new ideas” for the field of language acquisition. First, we recognize that researchers in the field of L2 pronunciation acquisition often need to conduct transdisciplinary research. Therefore, a three-layer conceptual model is used to introduce the existing literature from multiple disciplines and can also be used by other researchers to organize literature during their transdisciplinary research. Moreover, we argue that it is important for future research to emphasize mutual communicative responsibility and investigate interventions for both L2 Learners and L1 Listeners to address their linguistic experiences, cultural competence, and communication strategies. Different from the unilateral effort to improve L2 Learners’ pronunciation, we believe such interventions are feasible and necessary for people who need to communicate with L2 Learners frequently. Most importantly, we highlight a population which has been understudied in the field: child bilingual learners of non-English languages. Previous research, even though focused on different populations or languages, provided guidance for researchers to examine child interlocutors’ attitudes to L2 pronunciation and acquisition, their phonological transfer and adaptation in a variety of L1 and L2 combinations, and their production and perception of L2 pronunciation. In the future, more studies are needed on non-English languages and the child population in the context of continued globalization and thriving bilingual education. By discussing these themes and gaps, we hope to raise awareness among not only researchers who are interested in language acquisition, but also educators, practitioners, and policymakers to better facilitate children’s pronunciation learning and bilingual communication.

## Author contributions

YL proposed the conceptual model through literature review and wrote the first draft of the manuscript. All authors contributed to the manuscript revision, read and approved the submitted version, and discussion sessions of the literature and model revision.

## Funding

This work was supported by the SSHRC Insight Grant (435–2017-1086) and the Vanier Canada Graduate Scholarship through SSHRC (CGV–163274). The SSHRC Insight Grant funded a series of studies that focused on speech production in children enrolled in second-language education programs, which involved all the authors. The Vanier scholarship funded the YL’s doctoral studies.

## Conflict of interest

The authors declare that the research was conducted in the absence of any commercial or financial relationships that could be construed as a potential conflict of interest.

## Publisher’s note

All claims expressed in this article are solely those of the authors and do not necessarily represent those of their affiliated organizations, or those of the publisher, the editors and the reviewers. Any product that may be evaluated in this article, or claim that may be made by its manufacturer, is not guaranteed or endorsed by the publisher.
